# The Prevalence of High Carcinogenic Risk of HPV Genotypes among HIV-Positive and HIV-Negative MSM from Russia

**DOI:** 10.1155/2021/6641888

**Published:** 2021-05-31

**Authors:** Ilia Beliakov, Maria Senina, Yuriy Tyulenev, Elena Novoselova, Viktor Surovtsev, Alexander Guschin

**Affiliations:** ^1^NextBio Ltd. Со., Moscow 111394, Russia; ^2^InterLabService Ltd., Moscow 115035, Russia; ^3^RUDN University, Moscow 117198, Russia; ^4^Moscow Scientific and Practical Centre of Dermatovenerology and Cosmetology, Moscow 119071, Russia

## Abstract

**Objective:**

Men who have sex with men (MSM) have a high risk of lifelong anal cancer caused by high-risk human papillomavirus (HR HPV) infections. The aim of this study was to investigate the prevalence of anal canal HR HPV infection among men who have sex with men (MSM) with and without HIV infection in Moscow (Russia). We evaluated associations of some HIV coinfections (HSV and CMV) and HPV distribution among MSM with and without HIV infection.

**Methods:**

Two groups of HIV-positive (*n* = 60) and HIV-negative (*n* = 60) MSM were evaluated in the study. Fourteen high-risk (HR) HPV types, HSV1/2, and CMV were investigated in men anal swabs.

**Results:**

HR HPVs were found with nearly the same frequency of 66.7% in both groups: HIV-positive and HIV-negative MSM. HIV-positive status was statistically associated with the presence of several (more than two) HPV types (*p*=0.044). The most prevalent HR HPV genotypes were HPV18, HPV16, HPV56, and HPV33 for HIV-positive MSM and HPV56, HPV51, HPV66, and HPV16 for HIV-negatives. We found a statistically significant association of five HR HPV types with HIV status of MSM: HPV16 (*p*=0.028), HPV18 (*p*=0.00006), HPV58 (*p*=0.003), HPV33 (*p*=0.019), and HPV39 (*p*=0.026). The frequency of HSV1 (1.7%) and HSV2 (10%) infections and CMV (3.3%) infection was evaluated in the group of HIV-positive MSM. The frequency of HSV1 (5%) and HSV2 (6.7%) infections and CMV (0%) infection was evaluated, as well, in the group of HIV-negative MSM.

**Conclusion:**

Multiple HPV genotypes were detected significantly more often than single HPV genotype in the group of HIV-positive MSM. According to our data, 25% of HIV-positive MSM have HPV39; this is the only one of the five types of HR HPV (16, 18, 58, 33, and 39) associated with this group of MSM that has not yet been included in the HPV vaccines available on the market.

## 1. Introduction

Human papillomavirus (HPV) infection is the most common sexually transmitted infection (STI) worldwide. Mucosal HPV types from the alpha-genus infect squamous stratified epithelium from the mucosal tissues, and some infections may persist for many years with the potential to cause cancer of the cervix, vulva, vagina, penis, oropharynx, anus, and rectum [1, 2]. The HPV-attributable anogenital cancer includes 8500 vulva, 12000 vagina 35000 anus (half occurring in men), and 13000 penis. Anal squamous cell cancer, biologically similar to cervical cancer, is rare, but its incidence is increasing [3]. Almost all cervical cancer and more than 80% of anal cancer are attributable to high-risk HPV (HR HPV) infection including HPV types 16, 18, 31, 33, 35, 39, 45, 51, 52, 56, 58, 59, 68, 73, and 82 [4] and are mainly associated with HPV16 and HPV18 [5, 6].

These high-risk genotypes of HPV were identified to be distributed with different prevalence in both women and men with lesions or abnormal cytology worldwide [4, 7, 8]. The prevalence of HPV infection in heterosexual men and MSM in the Russian population was reported previously [9–11]. Also, infection of HPV and HSV I/II was evaluated in people living with human immunodeficiency virus or having high-risk sexual behavior including MSM from Russia [12, 13]. Comparison of HPV genotype prevalence and association with HIV status of MSM was previously reported in several populations [8, 14, 15]. However, there are no estimates of the prevalence of anal HPV and the distribution of high-risk HPV genotypes among HIV-negative and HIV-positive MSM from Russia [10, 11].

The HPV vaccines have been shown to be effective in preventing HPV infection and HPV-related low- and high-grade squamous intraepithelial lesions and cancer among both women and men [16, 17], and the 9-valent HPV vaccine has been shown to have a significantly increased potential impact compared with the quadrivalent vaccine among MSM [18]. The spectrum of high prevalent genotypes of HPV may be different in HIV-positive and HIV-negative MSM from Russia and influences the vaccine development [10, 11, 13].

HPV vaccination (bi- and quadrivalent HPV vaccines) is currently approved only for women in Russia. MSM do not receive the same benefits as heterosexual men from the herd effect of immunity from HPV vaccinations in girls [19, 20]. HPV vaccination is important among previously unvaccinated HIV-positive MSM aged up to and including 45 years [21–23].

## 2. Methods

### 2.1. Study Design

A cross-sectional study was conducted in Moscow, Russia, from November 2018 to October 2019. Participants were recruited by the nonprofit public organization “Step Fund” observed at the Moscow City Center for the Prevention and Control of AIDS and the Moscow Regional CDC. This study was approved by the Ethics Committee of the Institutional Review Board of the University of Peoples' Friendship University (RUDN University) of Russia, and written informed consent was obtained from each participant.

### 2.2. Data Collection

Participants were interviewed by well-trained health staff, and our special questionnaire was used to collect epidemiological data. Sociodemographic characteristics including age, past STIs (HPV and genital herpes), and CMV coinfection were collected. Participants were also asked about their behavioral characteristics including sexual orientation, sexual risks, and sexual behaviors.

### 2.3. Sample Collection and Laboratory Tests

Participants were asked to self-collect biomaterial from the anal canal using a regular flocked swab as described previously [11, 24] and store it in a universal transport media (NextBio, Russia) at −20°C.

Total DNA extraction was performed on 200 *μ*l of a sample using the MagnoPrime FAST kit (NextBio, IVD, Russia) on the Microlab STARLet platform (Hamilton Bonaduz AG, Switzerland) and eluted in 100 *μ*l TE buffer. 10 *μ*l of each DNA sample was analyzed using the AmpliPrime HR HPV genotyping system (NextBio, IVD, Russia), allowing for the detection of 14 high cancer risk HPV genotypes (HPV 16, 18, 31, 33, 35, 39, 45, 51, 52, 56, 58, 59, 66, and 68).

HSV and CMV DNA detection was performed in DNA from anal swabs of MSM participants both with AmpliPrime HSV/CMV kit (NextBio, IVD, Russia) and with reference kit AmpliSens HSV I, II-FL (Central Research Institute of Epidemiology, IVD, Russia).

### 2.4. Statistical Analyses

Statistical analysis was evaluated with suitable statistical criteria. Fisher exact test and chi-squared test were used to compare the distribution of categorical variables. A two-sided *p* value <0.05 was considered statistically significant.

## 3. Results

### 3.1. Sociodemographic Characteristics

A total of 60 HIV-positive and 60 HIV-negative MSM participants were included in the study. The median age was 29 years (interquartile range (IQR), 22–33.5) for HIV-positives and 33 years (IQR: 26–34) for HIV-negatives (Table 1).

### 3.2. Sexual Orientation

The HIV-positive participants declared their sexual orientation as homosexual (73.3%) and bisexual (26.6%). The sexual orientation reported by HIV-negative MSM was 68.3% homosexual and 31.6% bisexual.

### 3.3. Prevalence of HPV Infection in HIV-Positive and HIV-Negative MSM

**Figure 1:**
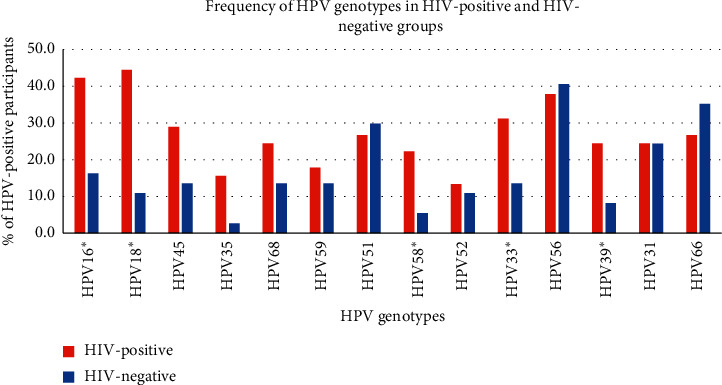
HPV genotypes in HIV-positive and HIV-negative MSM (*N* = 120). Asterisk marks the significant correlation of HPV genotype and HIV status.

Overall, the prevalence of high-risk HPV types was 73.3% among HIV-positive participants and 61.7% among HIV-negative men (*p*=0.18) (Table 1). We found that multiple HPV types were not significantly more frequent in the group of HIV-positive MSM (*p*=0.2). HPV genotyping data are presented in Figure 1.

Asterisk marks the positive correlation of HPV genotype and HIV status. The Fisher exact test analysis of HPV genotyping data revealed the significant association of five HR HPV genotypes with the HIV-positive status of the participants (Figure 1): HPV16 (*p*=0.028), HPV18 (*p*=0.00006), HPV58 (*p*=0.003), HPV33 (*p*=0.003), and HPV39 (*p*=0.026). For other HPV genotypes, no significant correlations were shown.

### 3.4. Age and HR HPV Infection Depending on the HIV Status

We analyzed HPV infection prevalence in the two age groups: <30 years and ≥30. Among our participants, the prevalence of anal HPV infection was the smallest within the 30- to 39-year interval (data not shown). The prevalence of any of HR HPV among HIV-positive participants was not significantly different: 44.2% (19/43) among those under 30 years old versus 56.8% (21/37) among the participants over 30 years (*p*=0.27) and increased with age similar to HIV infection: 45.1% (MSM under 30) vs. 57.1% (MSM over 30) (Table 1).

### 3.5. HSV and CMV Testing

HSV I/II was found in 11.7% (7/60) of HIV-positive participants (HSV I: 1/60, 1.7%; HSV II: 6/60, 10%) and in 11.7% (7/60) of HIV-negative (HSV I: 3/60, 5%; HSV II: 4/60, 6.7%). CMV DNA was identified in two HIV-positive participants (2/60, 3.3%), and no CMV was detected in HIV-negative participants.

## 4. Discussion

In this study, we showed the prevalence of anal HPV infection, genotype distribution, and the risk factors associated with the infection among MSM with or without HIV from Moscow, Russia. The average age in the two groups of our study was nearly equal (29.8 vs. 28.7 years). This study results confirmed data obtained from similar observations on the MSM population from Holland [25] and Russia [11]—HIV infection correlates with HPV infection.

HSV and CMV are known as the most frequent opportunistic infections among HIV-positive people, especially among MSM. It was previously shown by different authors HSV I/II and CMV coinfection in HIV-positive and HIV-negative MSM [26, 27]. Our data revealed a lower rate of HSV 1 coinfection in HIV-positive MSM in comparison with those in HIV-negative MSM (HSV1: 1.7% vs. 5%), and CMV was detected only in the HIV-positive group.

On the other side, the HIV-positive MSM tended to have more sexual partners and more sex episodes per lifetime than HIV-negative MSM [25]. We observed nearly equal sexual activity with both criteria: “a number of anal sex partners in the last 6 months” and “a number of lifetime male sex partners” in HIV-positive and HIV-negative groups. No significant correlation in sexual activity of MSM was observed in HIV-positive and HIV-negative groups. That might be due to the small number of participants in our study. It should be noted that the study participants had no health complaints at the moment of data and sample collection while CMV infections in the colon are common in HIV-infected people, often accompanied by anal ulcers in MSM.

It was found that HPV infection was associated with MSM and was detected with a high frequency (67.7%) regardless of HIV status. The most prevalent HR HPV genotypes reported in this study were HPV18 (33.3%), HPV16 (31.7%), HPV56 (28.3%), and HPV33 (21.7%) for HIV-positive MSM and HPV51 (20%), HPV56 (23.3%), and HPV66 (18.3%) for HIV-negative MSM. HPV16/18 previously showed 31.7% among MSM [11] compared to our data (40.8% (49/120) of all MSM tested). HPV16/18 were detected in the HIV-negative MSM group in 16.7% in the present study vs. 23% in the Wirtz study, and in the HIV-positive group, HPV16/18 were observed in 65% in our study vs. 41.4% in the Wirtz study [11].

We revealed association of five high-risk HPV genotypes with HIV status of MSM: HPV16 (*p*=0.028), HPV18 (*p*=0.00006), HPV58 (*p*=0.003), HPV33 (*p*=0.019), and HPV39 (*p*=0.026). One of the reasons for the correlation between the detection of HPV coinfection as previously published was the lower rate of HPV clearance in HIV-positive men [28, 29].

The higher rate of HPV associated with anal cancer incidence worldwide was observed among HIV-positive MSM (46 per 100,000 per year) compared to HIV-negative MSM (5 per 100,000 per year) [8, 24, 28].

There were several limitations to the current study. First of all, the selection of the study participants was not random. Due to the limitation of time of the study, we restricted the number of participants to 60 HIV-positive MSM and also randomly chose an equal number of participants from a bigger HIV-negative group. Second, the risk behaviors were measured based on self-report, which might lead to information bias. Third, although health professionals were trained using a standard protocol, the self-collection of anal canal cell samples might not be completely successful, and the test kit for HPV included 14 genotypes, which might together lead to an underestimation of the prevalence.

Cancer prevention of HIV-positive MSM highly depends on HR HPV vaccination. The majority of anal cancer cases associated with HPV 16/18 types were underrepresented in HIV-positive MSM diagnosed with anal cancers. Only several or few studies were published for now about HPV prevalence in anal cancer among HIV-positive MSM (HPV16: 67%; HPV18: 24%; HPV 31/33/45/52/58: 26%) and more thorough investigations of HPV genotypes should be performed on MSM anal cancers [15, 30]. HPV16/18-positive, HIV-positive MSM were detected in 48.3% while HPV16/18-positive, HIV-negative MSM were detected in only 20% in our study. Probably, HPV unvaccinated MSM more frequently coinfected both HPV and HIV. For the current vaccines present on the market, the nine-valent vaccine can protect from nearly 100% of described previously HR HPV in anal cancers of MSM (for cardasil9: 16, 18, 31, 33, 45, 52, and 58). We revealed a statistically significant association of five HR HPVs infections with HIV-positive MSM: HPV16, HPV18, HPV58, HPV33, and HPV39 in this study population.

HPV vaccination is not approved for men in Russia [16–18]: the 4-valent Gardasil (HPV 6, 11, 16, and 18) (Merck) vaccine is routinely prescribed for women vaccination and the nine-valent vaccine is not yet approved in Russia. The only HPV39 was not included in vaccines available on the market. According to recent data, about 7% of HIV-positive anal carcinomas contain HPV39 genotype [30]. This is an important issue that requires further detailed study: HPV vaccination in young and old MSM for the prevention of cancer associated with HPV [19] in Russia.

## Figures and Tables

**Table 1:** Characteristics of the study population (*n* = 120) of HIV-positive and HIV-negative MSM from Russia (2016–2017).

Influence of risk factors on HIV-infection		Total (*n* = 120)		HIV-negative (*n* = 60)		HIV-positive (*n* = 60)		*p* value
		No.	%	No.	%	No.	%	
Demographic characteristics								
Median age (IRQ)^#^		30 (24.5–34)		33 (26–34)		29 (22–33.5)		
Age group	<30	71	59.2	39	65	32	53.3	0.2
	≥30	49	40.8	21	35	28	46.7	

Sexual behavior								
No. of lifetime male sex partners								
No. of lifetime male sex partners	<100	47	41	26	47	21	35	0.16
	100–500	64	54	32	50	32	58	0.38
	>500	9	9	2	8	7	10	0.69

Median no. of anal sex partners last 6 months								
No. of anal sex partners last 6 months	≤1	22	18	10	17	12	20	0.35
	2–5	46	38	25	42	21	35	0.46
	≥6	52	43	25	42	27	45	0.71

Study results								
HPV infection vs HPV-negative		80	66.7	40	66.7	40	66.7	1
Multiple^*∗∗*^ HPV infection		60	50	34	56.7	26	43.3	0.044^*∗*^
Single HPV infection		20	16.7	6	10	14	23.3	
HPV16		27	22.5	8	13.3	19	31.7	0.028^*∗*^
HPV18		23	19.2	3	5	20	33.3	0.00006^*∗*^
HPV58		13	10.8	2	3.3	11	18.3	0.003^*∗*^
HPV33		18	15	5	8.3	13	21.7	0.019^*∗*^
HPV39		15	12.5	4	6.7	11	18.3	0.026^*∗*^
HIV-positive MSM within age groups	<30	71	59.2					
	≥30	49	40.8					
Any HR HPV-positive within age groups	<30	43	70.4	24	55.8	19	44.2	0.27
	≥30	37	63.3	16	43.2	21	56.8	

Co-infections								
HSV		14	11.7	7	11.7	7	11.7	1
HSV I		4	3.3	3	5	1	1.7	0.37
HSV II		10	8.3	4	6.7	6	10	0.5
CMV		2	1.7	0	0	2	3.3	—

IQR: interquartile range. ^*∗*^A two-sided *p* value <0.05 was considered statistically significant; ^*∗∗*^multiple HPV: two and more HPV types.

## Data Availability

The data used to support the findings of this study are available from the corresponding author upon request.

## Abbreviations

HR HPV: High-risk human papillomavirus
MSM: Men who have sex with men
STI: Sexually transmitted infection
IVD: In vitro diagnostic
HSV: Herpes simplex virus
HIV: Human immunodeficiency virus
CMV: Cytomegalovirus
AIN: Anal intraepithelial neoplasia.
